# Inflammaging and neurovascular unit dysfunction in cognitive ageing: mechanisms, biomarkers, and therapeutic opportunities

**DOI:** 10.3389/fnagi.2026.1841958

**Published:** 2026-07-13

**Authors:** Fan Bu, Shulin Zeng, Zhengchi Lou, Sijia Zhou, Xiufen Yang, Yi Wen, Weixiang Luo, Lan Qin

**Affiliations:** 1 Department of Neurology, Dongzhimen Hospital Affiliated to Beijing University of Chinese Medicine, Beijing, China; 2 Department of General Surgery, Qidong Hospital of Traditional Chinese Medicine, Qidong, Jiangsu, China; 3 Department of Traditional Chinese Medicine, The Third Affiliated Hospital of Henan Medical University, Xinxiang, Henan, China; 4 Department of Hepatobiliary Surgery, Shenzhen People’s Hospital (The First Affiliated Hospital Southern University of Science and Technology; The Second Clinical Medical College, Jinan University), Shenzhen, Guangdong, China; 5 Department of Emergency Medicine, Shenzhen People’s Hospital (The First Affiliated Hospital Southern University of Science and Technology; The Second Clinical Medical College, Jinan University), Shenzhen, Guangdong, China; 6 Department of Thoracic Surgery, Shenzhen People’s Hospital (The First Affiliated Hospital Southern University of Science and Technology; The Second Clinical Medical College, Jinan University), Shenzhen, Guangdong, China; 7 Department of Nursing, Shenzhen People’s Hospital (The First Affiliated Hospital Southern University of Science and Technology; The Second Clinical Medical College, Jinan University), Shenzhen, Guangdong, China; 8 Department of Neonatology, Shenzhen People’s Hospital (The First Affiliated Hospital Southern University of Science and Technology; The Second Clinical Medical College, Jinan University), Shenzhen, Guangdong, China

**Keywords:** blood-brain barrier, cerebrovascular reserve, cognitive ageing, inflammaging, neurovascular coupling, neurovascular unit

## Abstract

Age-related cognitive decline is increasingly recognised as arising from interacting immune and neurovascular vulnerabilities rather than from isolated insults. In this Review, we synthesise current human and mechanistic evidence within a convergence framework in which inflammaging and neurovascular unit (NVU) dysfunction are conceptualised as a feedback-coupled system. Persistent immune remodelling may promote endothelial activation, redox imbalance, pericyte–astrocyte support failure, microglial priming, and impaired neurovascular coupling, thereby reducing cerebrovascular reserve and destabilising blood–brain barrier (BBB) selectivity and transport. Conversely, emerging NVU dysfunction may amplify neuroimmune signalling and increase the susceptibility of white matter and synapses to late-life injury. We organise the literature around three mechanistic interfaces—immune–endothelium, glia–vascular, and flow–metabolic—and assess how this framework may clarify biomarker stratification, mechanism-aligned endpoint selection, and heterogeneity of intervention responses. Current evidence is strongest for integrative plausibility across inflammatory, vascular, and cognitive domains, but remains more limited for temporal ordering, effect modification, and reversibility in humans. We highlight key uncertainties, including peripheral-to-central signalling, harmonisation of BBB metrics, and heterogeneity across mixed late-life pathologies, and assess the therapeutic implications of immune- and NVU-directed strategies.

## 1 Introduction

Population ageing is increasing the prevalence of domain-specific cognitive slowing and executive dysfunction that meaningfully affect independence and quality of life. In later life, cognitive decline often reflects systems-level vulnerability and mixed pathophysiology rather than a single dominant lesion, arising from interacting immune, vascular, metabolic, and neural constraints that reduce resilience and increase sensitivity to secondary stressors (Santisteban and Iadecola, 2025). This heterogeneity is a major reason why late-life cognitive decline is difficult to understand through disease labels alone. Individuals with similar cognitive phenotypes may differ substantially in inflammatory burden, vascular reserve, BBB function, metabolic state, and neurodegenerative pathology, while apparently comparable biomarker profiles can be associated with very different cognitive trajectories depending on the integrity of compensatory systems (He and Sun, 2025).

A reproducible feature of ageing is a shift in baseline immune tone, often referred to as inflammaging: a persistent change in innate immune set-points, inflammatory resolution capacity, and stress reactivity. This state is shaped by immune remodelling across the lifespan and may be amplified by cellular senescence and senescence-associated secretory phenotype (SASP) signalling, cumulative metabolic stress, latent infection burden, and barrier–microbiome perturbations (Andonian et al., 2025). Yet the translational challenge is not simply that inflammatory signalling is elevated. Rather, the problem is that common blood readouts incompletely capture the immune programmes most relevant to endothelial and NVU function. This creates a recurring construct mismatch: the markers most convenient to measure are not necessarily those most informative about how ageing-related immune tone reshapes neurovascular physiology (Zeng et al., 2024).

In parallel, ageing erodes the buffering capacity of the neurovascular unit (NVU), the functional reserve that supports BBB selectivity and transport, microcirculation, neurovascular coupling, metabolic exchange, and clearance (Rowsthorn et al., 2023). Age-related changes in endothelial phenotype and transport, reduced pericyte support, impaired vasoreactivity, and coupling inefficiency can constrain the brain’s ability to match energy delivery to neuronal activity (Cunha et al., 2024). Because processing speed and executive control depend heavily on distributed network efficiency and white-matter integrity, reduced vascular reserve and coupling provide a plausible substrate for common cognitive ageing phenotypes (Groh and Simons, 2025). These changes may remain clinically subtle for long periods, becoming visible only when inflammatory, haemodynamic, or metabolic stress narrows compensatory margin further.

A central question emerging from current ageing research is whether inflammaging and NVU dysfunction are best understood as parallel correlates or as components of a feedback-coupled system. Across human, imaging, and mechanistic studies, the latter interpretation is increasingly plausible, although direct evidence for temporal ordering and interaction in humans remains incomplete (van Dinther et al., 2024). Within this framework, elevated immune tone may compress vascular reserve, bias endothelial phenotype, and destabilise BBB selectivity, while early NVU dysfunction may amplify neuroimmune signalling by increasing exposure to peripheral mediators and altering perivascular communication (Mekhora et al., 2024). This perspective helps explain why inflammation–cognition associations vary across cohorts, why ostensibly similar interventions yield heterogeneous effects, and why studies that measure only one axis may underestimate interaction or misclassify vulnerability.

This Review has three aims: to critically integrate inflammaging and NVU dysfunction within a single convergence framework; to evaluate the evidence across three mechanistic interfaces—immune–endothelium, glia–vascular, and flow–metabolic—with corresponding readouts; and to assess how far current evidence supports a pragmatic two-axis biomarker approach for stratification, endpoint selection, and interpretation of heterogeneous intervention responses. Neurodegenerative and vascular disorders are used as illustrative contexts rather than as disease-by-disease catalogues, with emphasis on mechanisms, measurable readouts, and translational interpretation grounded in construct clarity.

### 1.1 Scope and key definitions

In this Review, cognitive decline in ageing refers to age-associated deterioration spanning normal cognitive ageing, subjective cognitive concerns, and mild cognitive impairment, without presuming a single disease-specific aetiology. We focus particularly on domain-specific trajectories, including processing speed and executive function versus memory, because these domains may map differently onto NVU reserve and coupling constraints in mixed-pathology late-life states (Du et al., 2025). Alzheimer’s disease, Parkinson’s disease-related cognitive impairment, and vascular cognitive impairment are used as illustrative contexts rather than as exhaustive disease categories.

Inflammaging is defined as a persistent shift in immune tone arising from age-related immune remodelling, characterised by altered baseline activation, impaired resolution, and heightened stress reactivity. It is treated as a systems-level state variable rather than as a single biomarker, and is distinguished from transient inflammatory fluctuations.

The NVU is conceptualised as an integrated functional module comprising endothelial cells, pericytes, astrocytes, microglia, neurons, vascular smooth muscle cells, and extracellular matrix elements. NVU dysfunction includes impaired BBB selectivity and transport, reduced microvascular reserve, impaired neurovascular coupling, altered metabolic exchange, and downstream white-matter vulnerability, including changes that may precede extensive MRI-visible structural damage.

**Figure 1:**
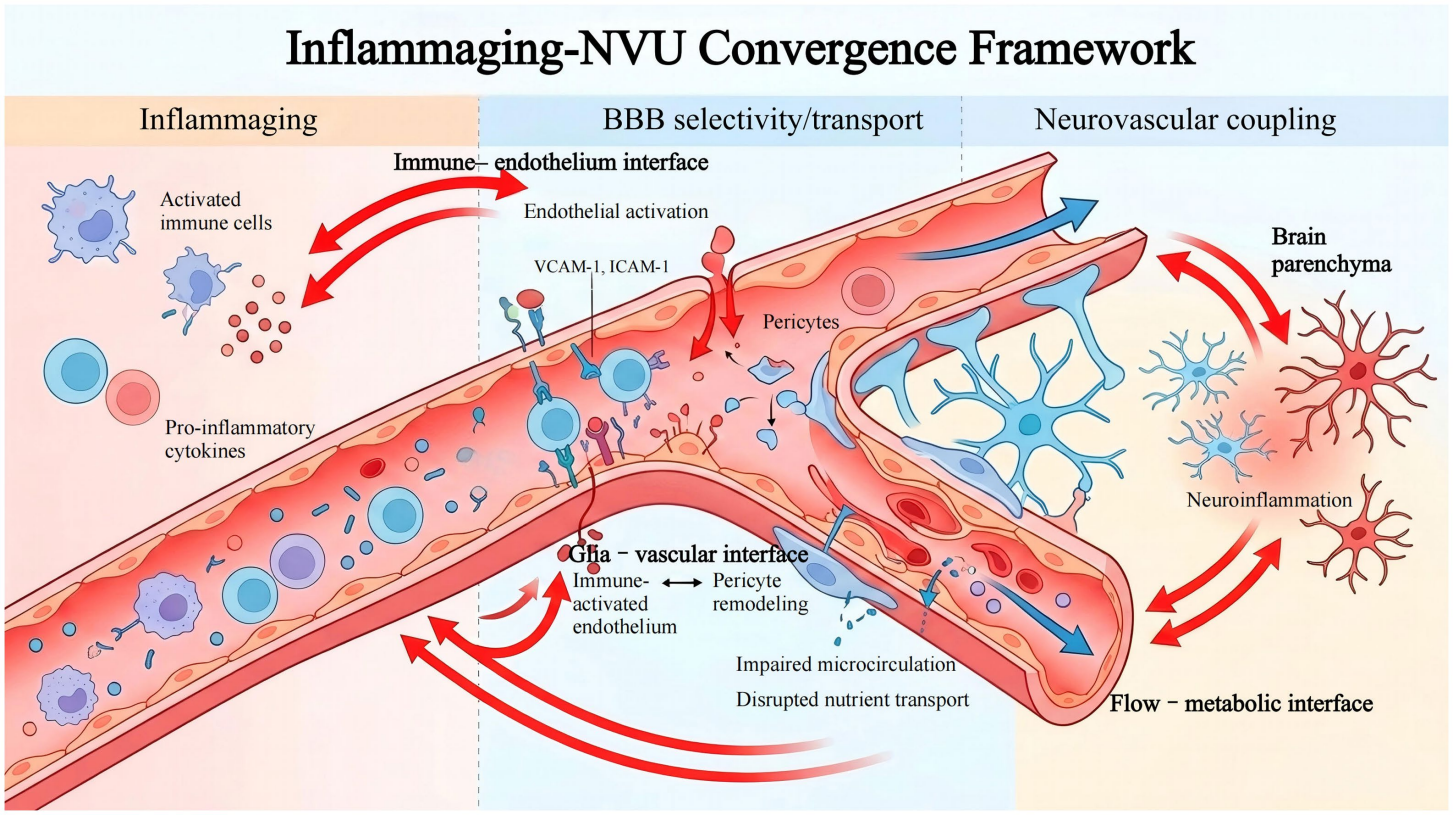
Convergence model linking inflammaging, neurovascular unit dysfunction, and cognitive ageing. Age-related immune remodelling and neurovascular unit (NVU) dysfunction are depicted as a feedback-coupled system linked through immune–endothelium, glia–vascular, and flow–metabolic interfaces. Chronic inflammatory tone may reduce vascular reserve, impair BBB selectivity/transport, and disrupt neurovascular coupling, whereas emerging NVU dysfunction may amplify neuroimmune signalling and increase white-matter and synaptic vulnerability. The model is heuristic and intended to guide mechanistic interpretation and biomarker stratification rather than to define fixed clinical categories.

Convergence is used in a mechanistic sense: inflammaging and NVU dysfunction may arise from shared upstream ageing programmes and may reciprocally amplify one another. Informative biomarkers and effective interventions may therefore need to represent, and in some settings target, both axes. Figure 1 summarises the proposed convergence model across the immune–endothelium, glia–vascular, and flow–metabolic interfaces.

## 2 Literature search and structured narrative synthesis

This Review was designed as a structured, framework-led narrative synthesis rather than a systematic review or meta-analysis. Its primary objective was to integrate heterogeneous evidence across mechanistic and translational domains and to develop a testable convergence framework linking inflammaging, neurovascular unit dysfunction, and cognitive ageing. The relevant literature spans human observational cohorts, vascular physiology, BBB-oriented imaging, interventional studies, and mechanistic animal and cellular models. These studies frequently assess non-equivalent constructs, use different protocols and timescales, and report heterogeneous cognitive and neurovascular endpoints. Accordingly, the current evidence base does not support a single defensible pooled effect estimate. We therefore used a transparent, framework-led approach to evidence identification, prioritisation, and cross-modal triangulation, while avoiding claims of exhaustive study-level coverage.

### 2.1 Evidence identification and prioritisation

We searched PubMed/MEDLINE, Embase, and Web of Science Core Collection. We also screened the reference lists of key articles and recent syntheses and searched ClinicalTrials.gov for ageing-relevant studies targeting inflammatory and/or neurovascular mechanisms. The search was updated to 31 January 2026, with no language or date restrictions.

#### 2.1.1 Search terms

Combined three concept blocks: (1) ageing, inflammaging, immune ageing, immunosenescence, and senescence-associated secretory phenotype signalling; (2) neurovascular unit, blood–brain barrier, neurovascular coupling, cerebrovascular reactivity, perfusion, small-vessel disease, and white matter; and (3) cognition, cognitive decline, mild cognitive impairment, and cognitive ageing. Targeted searches were also undertaken for specific NVU constructs, including BBB selectivity, transport and permeability measures, perfusion-related measures, cerebrovascular reactivity, neurovascular coupling, and emerging clearance-adjacent metrics.

We prioritised studies that jointly considered inflammatory tone, NVU-related measures, and cognitive outcomes; used longitudinal or multimodal designs; or provided interventional leverage relevant to directionality or therapeutic timing. Mechanistic animal and cellular studies were included when they provided plausible bridges across the immune–endothelium, glia–vascular, or flow–metabolic interfaces.

### 2.2 Evidence weighting and structured synthesis

Human longitudinal and interventional evidence was given the greatest weight for inference regarding timing, clinical relevance, and potential reversibility. Cross-sectional human studies were interpreted primarily as hypothesis-generating, while mechanistic animal and cellular studies were used to assess biological plausibility and identify intermediate phenotypes that may be operationalised in human studies. Where possible, we prioritised studies that excluded acute inflammatory events at sampling, used repeated inflammatory measures, or incorporated multimodal phenotyping. Negative or mixed findings were retained when they challenged assumptions regarding peripheral-to-central correspondence or highlighted limitations in construct validity.

**Figure 2:**
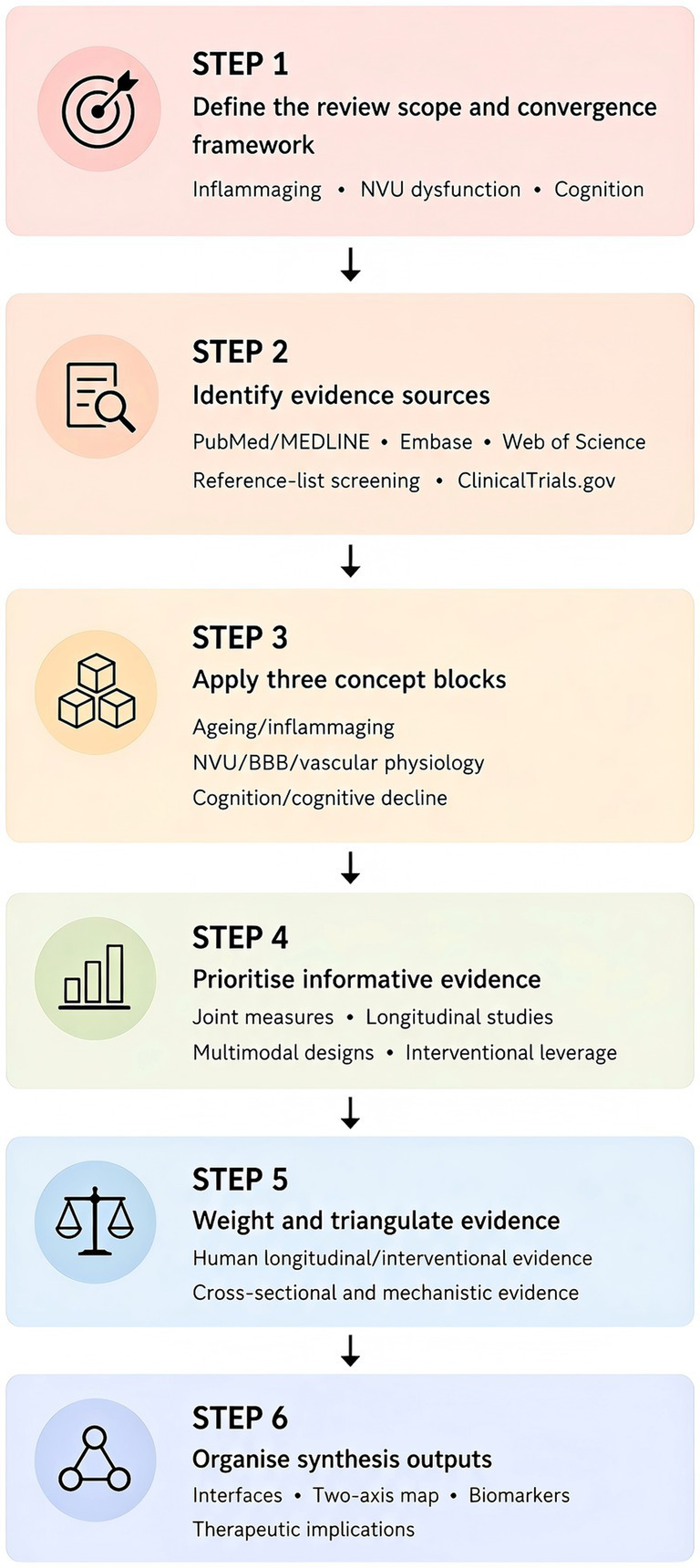
Framework-led workflow for evidence identification and structured narrative synthesis. This schematic illustrates the framework-led workflow used for evidence identification, prioritisation, weighting, and synthesis. It is intended to clarify the structured narrative approach and should not be interpreted as a PRISMA study-selection flow diagram. BBB, blood–brain barrier; NVU, neurovascular unit; SASP, senescence-associated secretory phenotype.

Across evidence types, interpretation was guided by evidence hierarchy and cross-modal triangulation rather than vote counting. Particular attention was paid to phenotypic heterogeneity, discordance across blood, CSF, imaging, and physiological measures, mismatch between acute inflammatory fluctuations and slower neurovascular remodelling, and survivor or sampling bias in imaging-rich cohorts. The framework-led workflow for evidence identification, prioritisation, weighting, and synthesis is summarised in Figure 2.

## 3 Conceptual framework

### 3.1 Definitions and boundaries

The literature reviewed here supports considering inflammaging and NVU dysfunction as components of a feedback-coupled, two-axis system that may accelerate cognitive decline in ageing, including in mixed-pathology states where conventional disease labels incompletely capture underlying biology (Anderle et al., 2025; Maldonado-Díaz et al., 2024).

Within this framework, inflammaging is treated as a systems-level state variable rather than a single pathway, while NVU dysfunction is defined functionally rather than anatomically. The latter includes impaired BBB selectivity/transport, reduced microvascular reserve, impaired neurovascular coupling, altered metabolic exchange, and impaired clearance, including changes that may precede extensive infarcts or white-matter lesions (Che et al., 2024; Moran et al., 2025). This functional definition prevents the framework from being narrowed prematurely to “vascular dementia” or overt small-vessel disease, both of which represent relatively late and heterogeneous manifestations of broader neurovascular compromise.

In this Review, convergence refers mechanistically to shared upstream ageing programmes that bias both immune tone and NVU vulnerability and/or to bidirectional amplification loops whereby inflammatory tone alters NVU function and NVU dysfunction sustains neuroimmune activation. The translational value of the framework would be strengthened if joint measurement of the two axes improves the explanation or prediction of cognitive trajectories relative to either axis alone (Mayer and Fischer, 2024).

This framework is disease-agnostic with respect to primacy. It accommodates neurodegeneration-dominant, vascular-dominant, and mixed scenarios. Its purpose is not to replace disease-specific models, but to provide an ageing-centred scaffold for understanding why cognitive decline may accelerate when immune and neurovascular vulnerabilities intersect. In this sense, convergence is not proposed as an alternative diagnosis. It is a way of organising mechanisms, measurements, and therapeutic logic across late-life conditions in which traditional categorical boundaries often obscure rather than clarify causal structure.

#### 3.1.1 Two-axis state space

Taken together, current evidence supports viewing convergence as a two-axis framework in which Axis 1 indexes immune tone or inflammaging burden and Axis 2 indexes NVU reserve, coupling, and BBB-related vulnerability (Bowie et al., 2024). Within this framework, cognitive decline is expected to be greatest when high inflammaging co-occurs with low reserve and impaired coupling, and responses to intervention are likely to depend importantly on baseline position across these two axes.

This two-axis framing is intended as a tractable abstraction rather than an exhaustive model of cognitive ageing. Its main value is to explain why inflammatory burden is not uniformly harmful, why NVU abnormalities may remain clinically silent, and why apparently similar interventions produce divergent outcomes. Both axes are likely to vary continuously, with risk depending on relative position and movement over time rather than on simple normal/abnormal categories.

### 3.2 Key interfaces linking inflammaging to NVU dysfunction

The convergence framework can be organised around three interfaces through which systemic immune ageing most plausibly engages neurovascular physiology. These interfaces are useful because each captures a coherent mechanism, maps onto measurable readouts, and offers potential entry points for stratification and endpoint selection (Oprea et al., 2025). They are not mutually exclusive. In most ageing phenotypes, more than one interface is likely to be engaged, with relative prominence varying by stage, comorbidity, and pathology mix.

#### 3.2.1 Immune–endothelium interface

Age-related inflammatory signalling can bias cerebral endothelium towards an activated phenotype, with consequences for BBB selectivity, transport function, and vascular responsiveness. Even in the absence of overt barrier leak, low-grade inflammatory tone may alter nitric oxide bioavailability, redox balance, and endothelial adhesion or trafficking programmes, thereby reshaping immune–vascular communication at the brain interface (Kovbasyuk et al., 2025).

Endothelium translates systemic signals into local neurovascular consequences through its roles in barrier regulation, vasomotor signalling, substrate exchange, leukocyte trafficking, and communication with mural cells and astrocytes. Persistent endothelial shifts may therefore have functional consequences before conventional imaging detects structural damage.

Readouts at this interface include endothelial activation and vascular inflammation signatures, BBB-related physiological measures where available, and haemodynamic measures such as cerebrovascular reactivity and perfusion.

#### 3.2.2 Glia–vascular interface

A second interface operates through glial regulation of vascular and barrier function. With ageing, microglia may become primed and astrocytes may remodel their perivascular endfeet, shifting the balance between inflammatory signalling and homeostatic support. Because glia participate in barrier maintenance, metabolic support, and regulation of vascular tone, changes in glial state provide a plausible route by which systemic inflammaging translates into altered NVU stability (Labarta-Bajo and Allen, 2025).

This interface bridges peripheral immune tone and local brain vulnerability without assuming direct correspondence between peripheral markers and central inflammation. Glial states may integrate prior exposure, local pathology, and the current systemic milieu, potentially sustaining NVU dysfunction even when peripheral inflammatory levels fluctuate.

Relevant readouts are typically multimodal, combining neuroimmune markers, coupling-related physiology, and downstream white-matter or network-level signatures.

#### 3.2.3 Flow–metabolic interface

The third interface concerns matching vascular supply to neural metabolic demand. Neurovascular coupling is a core NVU function that depends on coordinated endothelial, mural-cell, and glial signalling. In ageing, coupling may decline through reduced endothelial responsiveness, altered vasoactive signalling, microvascular rarefaction, haemodynamic stiffening, and glial remodelling (Khartabil et al., 2026).

Because this interface lies close to function, its readouts are particularly attractive for stratification and intermediate endpoints. These include cerebrovascular reserve, perfusion efficiency, and coupling measures that relate flow to neural activity. Importantly, flow–metabolic failure need not imply severe baseline hypoperfusion. The clinically relevant issue is often dynamic insufficiency: the inability to deliver appropriately timed, regionally targeted increases in blood flow during cognitive demand.

The flow–metabolic interface may also be especially relevant to the cognitive domains most consistently affected in normal ageing, because executive control, attention, and processing speed depend on the integrity of widely distributed and energetically demanding networks (Li et al., 2024).

The principal testable predictions, falsifiers, and minimum measurement logic of the proposed convergence model are summarised in Box 1.

Box 1 **Testable predictions and falsifiers for the inflammaging–NVU convergence model**

*Conceptual axes.*

Axis 1 (inflammaging burden): stable or repeated inflammatory composites, innate–adaptive immune remodelling indices, and senescence-associated signalling proxies.

Axis 2 (NVU vulnerability): vascular reserve/coupling together with BBB selectivity/transport, distinct from leak alone (Uchida et al., 2023).

*A. Core predictions.*

P1. Interaction (effect-modification) prediction.

Prediction: The association between inflammaging burden and cognitive decline is strongest when NVU vulnerability is high, and weakest when vascular reserve, coupling, and BBB selectivity are preserved.

Falsifier: Interaction terms are consistently null across well-powered cohorts using harmonised NVU measures, with only independent main effects observed.

P2. Temporal-ordering prediction.

Prediction: Rising inflammaging precedes, or co-evolves with, progressive loss of vascular reserve/coupling and BBB selectivity/transport changes, which in turn precede steeper cognitive decline, especially in mixed-pathology phenotypes.

Falsifier: Longitudinal studies with adequate sampling density show NVU decline without antecedent or parallel changes in inflammaging, or inflammaging shifts without subsequent NVU change.

P3. BBB selectivity/transport over leak prediction.

Prediction: In non-demented older adults, BBB dysfunction relevant to cognitive ageing is better captured by selectivity/transport impairment than by permeability or leak measures alone (Denkinger et al., 2024).

Falsifier: Leak-based measures fully account for cognitive associations and treatment responsiveness, with selectivity/transport measures adding no explanatory value.

P4. Interface-specific signature prediction.

Prediction: Distinct inflammatory–NVU signatures should map onto the three mechanistic interfaces: immune–endothelium, glia–vascular, and flow–metabolic.

Falsifier: Markers do not cluster reproducibly by interface and show inconsistent directionality across studies.

P5. Phenotype-guided intervention prediction.

Prediction: Older adults with both high inflammaging and high NVU vulnerability derive the greatest benefit from combined or sequenced strategies that reduce inflammatory tone and improve NVU reserve/coupling or BBB transport.

Falsifier: Single-axis interventions perform equally well across phenotypes, with no enrichment advantage in the high–high quadrant.

*B. Minimum measurement logic.*

Testing the convergence model requires representation of both axes: repeated inflammatory measures to approximate persistent immune tone and at least one function-proximal NVU measure, such as cerebrovascular reactivity or neurovascular coupling. BBB selectivity/transport measures and structural injury markers provide additional mechanistic and staging information where feasible (Yang et al., 2024). Minimum measurement sets for different study designs are summarised in Table 1.

*C. Practical implication.*

Support for the framework would be strengthened by concordant change in at least one immune measure and one NVU endpoint within the expected biological time window. Isolated change in one axis may reflect construct mismatch, inadequate timing, insufficient intervention intensity, or incorrect phenotype assignment.

## 4 Inflammaging

### 4.1 Origins and amplifiers of inflammaging relevant to brain ageing

Inflammaging reflects layered, age-dependent shifts in immune regulation rather than a single upstream trigger (Thi Quynh Trang and Kyung, 2025). Ageing reshapes immune set-points, particularly within innate and myeloid compartments, reduces adaptive flexibility, and weakens resolution programmes (Shuaiqi et al., 2025). For brain ageing, the key implication is not simply higher cytokine concentrations, but a persistent bias in immune tone that renders endothelial and glial systems easier to prime and harder to return to baseline (Wenyan et al., 2024).

Several amplifiers are especially relevant to cognitive trajectories and NVU vulnerability. Cellular senescence and SASP signalling can provide persistent pro-inflammatory cues (Linlin et al., 2026). Metabolic stress, including insulin resistance, visceral adiposity, and mitochondrial dysfunction, may sustain inflammatory tone and oxidative stress while intersecting directly with endothelial NO/redox balance and coupling efficiency (Xianhong et al., 2025). Barrier–microbial factors, including gut permeability and microbiome shifts, may add intermittent inflammatory stimuli that become more consequential on an aged immune background (Alison et al., 2024). Additional contributors such as chronic latent infection burden, altered sleep architecture, and frailty-related physiological instability may further magnify the persistence or reactivity of inflammatory programmes (Christina et al., 2024; Yiming and Lina, 2024).

A central translational need is therefore to distinguish stable inflammaging from acute inflammatory events and to identify signatures that are mechanistically coupled to endothelial and NVU constraints rather than reflecting comorbidity alone (Aline et al., 2025). This distinction is clinically important. An older adult with elevated inflammatory markers after a recent infection, poor sleep, or medication change may not occupy the same biological state as an older adult with stable, repeated evidence of chronic immune activation. Yet both may appear similar in a cross-sectional dataset. Repeated sampling, trajectory modelling, and attention to co-occurring metabolic and vascular context are therefore especially important when attempting to relate immune tone to neurovascular and cognitive outcomes (Caldeira et al., 2025; Cai et al., 2025).

### 4.2 Inflammatory programmes most relevant to the NVU

Not all inflammatory activity is equally informative for understanding neurovascular dysfunction in ageing. Within the convergence framework, the most relevant inflammatory programmes are those that plausibly alter endothelial phenotype and BBB selectivity/transport, modulate glial states involved in coupling and homeostasis, or generate redox and metabolic constraints that compress vascular reserve.

#### 4.2.1 Endothelial-activating cytokine networks

Chronic low-grade inflammatory signalling can bias endothelium towards activation, altering BBB selectivity and transport without necessarily causing overt leak, increasing adhesion and trafficking capacity, and reducing vasodilatory reserve through NO and redox constraints (Abbatecola et al., 2024). Functionally, these shifts may degrade perfusion efficiency and neurovascular coupling while facilitating sustained immune–brain communication (Kim and Cheon, 2024). Because these pathways sit at the intersection of systemic inflammation and brain access, even subtle endothelial activation may determine whether similar inflammatory burden remains cognitively silent or becomes clinically relevant.

#### 4.2.2 Innate immune amplification and inflammasome-linked signalling

Ageing commonly favours innate amplification loops that translate sterile danger signals, including metabolic stress, mitochondrial dysfunction, and cellular debris, into persistent inflammatory output (Scarpellino et al., 2024). Within the NVU, this may contribute to endothelial dysfunction, glial priming, and impaired coupling, particularly when reserve is already constrained by vascular stiffening and microvascular remodelling (Liang et al., 2024). These programmes are especially relevant because they blur the distinction between inflammation and tissue stress, linking frailty, cardiometabolic dysregulation, and brain vulnerability through chronically raised baseline set-points rather than dramatic inflammatory surges.

#### 4.2.3 Complement and opsonisation programmes

Immune pathways involved in tagging and clearance can influence brain function through microglial surveillance and synaptic support (Peng et al., 2025). With ageing, dysregulated activation may increase vulnerability to maladaptive pruning and exaggerated responses to injury or pathology. In the convergence framework, the translational importance of these pathways lies in their ability to couple glial-state changes to vascular regulation and barrier maintenance. In mixed late-life states, complement-related biology may help explain why inflammatory burden aligns more closely with network inefficiency or structural vulnerability than with overt neurodegenerative biomarkers alone (Negro-Demontel et al., 2024).

#### 4.2.4 Oxidative stress and redox–inflammation coupling

Chronic inflammation and oxidative stress reinforce one another. Redox imbalance may reduce endothelial NO bioavailability, impair vasoreactivity, and exacerbate mitochondrial stress in NVU-associated cells (Nimmo et al., 2024). Because coupling depends on finely regulated vasoactive signalling and metabolic support, redox–inflammation coupling provides a compact route by which inflammaging may compress functional reserve and accelerate decline in cognitively demanding networks. This mechanism may help explain why inflammatory burden is often linked more clearly to executive and processing-speed decline than to isolated memory phenotypes (Neyra Chauca et al., 2026).

### 4.3 Evidence strength and uncertainties

Human evidence is strongest for associations between inflammatory tone and vascular or NVU-related readouts, whereas mechanistic specificity and directional inference still rely heavily on preclinical and cellular studies. Peripheral inflammatory signatures may also map imperfectly onto CSF, imaging, or physiological readouts, arguing for multimodal inference rather than single-marker narratives (Ungvari et al., 2025).

### 4.4 Translational bottlenecks: why “anti-inflammatory” does not automatically translate into cognitive benefit

A persistent paradox is that inflammaging is associated with worse cognitive trajectories and greater NVU vulnerability, yet anti-inflammatory strategies often yield mixed cognitive outcomes in older adults (Giri et al., 2024). Four bottlenecks are especially relevant.

First, construct mismatch: many interventions lower a subset of peripheral markers without affecting the programmes that constrain endothelial function, BBB selectivity/transport, or neurovascular coupling. A reduction in a convenient blood marker does not necessarily indicate that the inflammatory programme most relevant to brain ageing has shifted (Mathot et al., 2025).

Second, timescale mismatch: inflammatory markers may shift over days to weeks, whereas NVU dysfunction and white-matter vulnerability often evolve over months to years. Mechanism-proximal physiological endpoints may therefore be more informative over short follow-up windows than cognition alone. A true biological effect may be missed when only distal outcomes are measured too early, or when early physiological change is discounted because global cognition remains stable (Gonzalez-Bautista et al., 2025).

Third, heterogeneity and mixed pathology: late-life populations vary widely in vascular burden, neurodegeneration, metabolic state, frailty, and medication exposure. Average effects are easily diluted without phenotype-informed stratification. Indeed, one reason inflammatory interventions have appeared inconsistent may be that they are evaluated across populations in whom the limiting biology differs substantially.

Fourth, safety and feasibility constraints: immunomodulation in older adults is limited by infection risk, impaired vaccine responses, multimorbidity, and polypharmacy, all of which can restrict intensity and duration of treatment. This creates a bias towards modest biological perturbation even when stronger engagement might be required (Hofer et al., 2025).

Taken together, these bottlenecks argue against treating “anti-inflammatory” as a coherent therapeutic class. Instead, they support a more mechanistically matched approach in which inflammatory modulation is interpreted alongside NVU physiology and cognitive trajectories. They also support a more restrained interpretation of null cognitive results: absence of short-term cognitive improvement after peripheral marker change does not, by itself, resolve the role of inflammaging in cognitive ageing. The major inflammatory–NVU interfaces, their representative human readouts, and their main evidentiary uncertainties are summarised in Table 2.

## 5 Ageing-driven NVU dysfunction

### 5.1 BBB/NVU ageing phenotype: barrier regulation and selective transport

Age-related neurovascular decline often begins as a functional phenotype rather than as an overt lesion. Within the NVU, the BBB is not a static wall but a regulated interface governing selective transport, metabolic exchange, and signalling between periphery and brain (Santisteban and Iadecola, 2025). In ageing, BBB dysfunction may therefore first present as shifts in selectivity and transport capacity, even when conventional measures do not detect frank permeability increase (Ruslan et al., 2025).

Mechanistically, age-related changes in endothelial phenotype, tight-junction regulation, adaptive transport capacity, and the perivascular microenvironment involving pericytes, astrocytic endfeet, and extracellular matrix can all shift BBB behaviour (Yating et al., 2025). These changes matter for cognition because transport inefficiency reduces buffering of systemic variability, while altered selectivity may increase exposure to peripheral mediators that prime glial responses and destabilise coupling (Devraj et al., 2024).

A useful translational distinction is between selectivity/transport changes and paracellular leak. These dimensions may overlap, but they are not identical, and studies should state clearly which construct is being indexed. Within a convergence framework, BBB alterations are most likely to matter when inflammatory tone is elevated and compensatory neurovascular reserve is constrained. This distinction matters because a leak-centred framing may bias both measurement and interpretation towards later or more overt barrier injury, whereas ageing-related cognitive vulnerability may be shaped earlier by subtler transport and interface dysfunction (Konig et al., 2025).

BBB ageing is also likely to be regionally heterogeneous, which may help explain selective cognitive signatures despite only modest global barrier dysfunction (Alaqel et al., 2025).

### 5.2 Microcirculation and vasoreactivity: perfusion efficiency and vascular reserve

Beyond barrier regulation, cognitive resilience depends on cerebral microcirculation and vascular reserve. Ageing is associated with reduced haemodynamic flexibility, often expressed as diminished vasoreactivity and weaker buffering of physiological challenges such as CO₂ fluctuations, postural change, or blood pressure variability (Weijs et al., 2024). The key problem is often not global hypoperfusion but reduced reserve and inefficient dynamic allocation during cognitively demanding states (You et al., 2023).

Microvascular inefficiency may arise through interacting processes that include endothelial NO/redox constraints, capillary rarefaction, altered flow dynamics, impaired pericyte-related capillary regulation, and increased transmission of pulsatility into distal microvessels (Kapoor et al., 2024). This fits well with cognitive ageing phenotypes dominated by processing speed and executive dysfunction, which depend on distributed network efficiency and white-matter integrity (Tap et al., 2023).

Within the convergence model, reserve functions as a buffer variable: its importance lies not only in correlating with cognition, but in helping explain when inflammatory tone translates into measurable functional decline. This buffer concept is important because reserve is likely to modify both acute vulnerability and longer-term slope. A person with reduced reserve may not simply perform worse at baseline; they may also be less able to tolerate transient inflammatory or haemodynamic perturbations, thereby accumulating injury over time (Álvarez-Bueno et al., 2024).

The clinical meaning of low reserve is therefore broader than vascular underperformance in a narrow sense. It marks a system operating closer to its limits, one in which small insults are more likely to become clinically relevant.

### 5.3 Neurovascular coupling and network vulnerability

Neurovascular coupling, the moment-to-moment adjustment of local blood flow to neuronal activity, is the NVU function closest to cognition. In ageing, coupling may become less efficient or less precise, turning cognitively demanding states into periods of relative energetic stress for vulnerable networks (Gildea and Liddelow, 2025). Because coupling is an emergent property of endothelial, mural-cell, and glial signalling, it provides a natural point of convergence for immune tone, endothelial health, and glial state (Divecha et al., 2025).

Coupling impairment may arise through reduced endothelial responsiveness, altered astrocyte-mediated regulation, microvascular flow heterogeneity linked to pericyte dysfunction, and glial states that prioritise inflammatory over homeostatic functions (Zeng et al., 2025). Cognitively, this may be expressed less as a single-domain deficit than as reduced network efficiency, fatigability, and stress sensitivity, particularly in executive control, attention, and processing speed (Hanna et al., 2024).

This functional proximity to cognition gives coupling particular translational appeal. It may emerge earlier than structural injury, but remains methodologically demanding and sensitive to baseline vascular state, CO₂, medication effects, and analysis choices. Coupling is also attractive translationally because it may serve as a mechanism-proximal endpoint when cognitive change is slow. When interventions shift inflammatory markers but fail to alter reserve or coupling in the predicted direction, construct mismatch, insufficient timing, or the need for dual-axis strategies become plausible interpretations (Sun et al., 2025).

### 5.4 White-matter vulnerability and small-vessel signatures

Although NVU dysfunction may be predominantly functional early on, ageing often leaves structural footprints that anchor the convergence model to observable brain change. White matter is especially relevant because long-range connectivity and processing speed depend on metabolically demanding myelinated tracts that are sensitive to microvascular insufficiency (Christopher et al., 2025).

At the same time, structural signatures are biologically non-specific and should not be treated as direct surrogates of a single mechanism. White-matter lesions and microstructural abnormalities reflect multiple upstream contributors, including vascular risk, immune tone, metabolic stress, and neurodegenerative cascades (Gu et al., 2025). Within the convergence model, structural burden is therefore best viewed as a stage marker: it identifies later accumulation, constrains expectations about reversibility, and should be interpreted alongside functional NVU readouts (Li et al., 2025b).

This stage-marker framing is especially important for intervention logic. Extensive structural burden does not falsify the relevance of inflammaging or NVU dysfunction; rather, it suggests that functional recovery may be limited or slow, and that stabilisation may be a more realistic goal than reversal. Conversely, when structural accumulation remains modest, reserve/coupling impairment may offer a clearer window for detecting biological engagement and for testing whether dual-axis strategies can alter trajectory before damage becomes entrenched (O’Sullivan, 2024).

## 6 Convergence of inflammaging and NVU dysfunction

### 6.1 Bidirectional amplification loops

The convergence framework is most informative when inflammaging and NVU dysfunction are considered as coupled state variables rather than as parallel correlates. Chronic inflammatory tone may compress the operating margin of neurovascular physiology by reducing reserve and coupling efficiency, while emerging NVU dysfunction may alter barrier selectivity and perivascular signalling in ways that sustain or amplify neuroimmune activation (Anderle et al., 2025; Sachdev et al., 2026).

The three interfaces described above provide the main contact surfaces through which this coupling may operate. Repeated perturbation, in the context of limited buffering capacity, may gradually convert functional vulnerability into cumulative structural burden, particularly in white matter (Beran et al., 2024). At the same time, barrier and perivascular changes may increase effective exposure to peripheral mediators, further biasing inflammatory set-points (Friedman et al., 2025).

This framing implies that associations between immune tone and cognitive decline should be strongest when reserve and coupling are constrained (Mekhora et al., 2024), that transient stressors should have disproportionate effects in low-reserve phenotypes (Chen T. et al., 2024), and that early changes should be detectable in reserve/coupling and BBB-related phenotypes before extensive structural accumulation dominates (Lennon et al., 2025).

An important feature of this model is that amplification need not be dramatic to be clinically important. Small, repeated mismatches between inflammatory load and neurovascular buffering may accumulate over years, especially in white matter and other regions dependent on efficient microvascular support (Siddiquee et al., 2025). In this sense, convergence is as much about chronic failure of recovery and compensation as about acute injury (Zhang et al., 2024).

### 6.2 Multilevel evidence for convergence: strengths and gaps

The literature currently supports convergence more strongly as an integrative and testable framework than as a fully validated human interaction model. A convincing human demonstration would ideally require repeated immune profiling, function-proximal NVU measures, and temporally ordered outcomes capable of testing interaction and reversibility. Much of the current literature only partially satisfies these conditions, leaving an evidentiary “missing middle” between mechanistic plausibility and definitive human validation (Markus et al., 2022; Rowsthorn et al., 2025).

#### 6.2.1 Human observational evidence

Across ageing cohorts, higher inflammatory tone is commonly associated with poorer cognition and steeper decline, with effects often more apparent in processing speed and executive domains than in isolated amnestic performance (Bahorik et al., 2024; Persin et al., 2024). In parallel, NVU-relevant measures, including microvascular burden, reduced reserve, perfusion inefficiency, impaired neurovascular coupling, and white-matter vulnerability, are also associated with cognitive trajectories (Yang and Webb, 2023; Liao et al., 2025). The convergence claim becomes stronger when studies move beyond single-axis association and show that multimodal or joint-marker approaches better capture risk architecture than isolated predictors alone (Lennon et al., 2025; Huang et al., 2025).

The main limitation is causal resolution. Peripheral inflammatory signatures may reflect multimorbidity, frailty, adiposity, medication exposure, and socioeconomic gradients, while NVU measures vary by modality, protocol, and physiological state. Observational evidence therefore supports the existence of a risk architecture compatible with convergence more strongly than it demonstrates a fully validated human interaction model. That distinction is important. It tempers causal claims while still allowing the framework to remain scientifically useful.

#### 6.2.2 Interventional and natural-experiment leverage

Convergence is more directly tested when an intervention shifts one axis while the other is tracked as a downstream or parallel response. At the population level, dementia-prevention frameworks consistently support the importance of vascular and lifestyle risk modification, but they also emphasise heterogeneity in timing, baseline risk, and measurable response (Livingston et al., 2020). In trial-oriented contexts, multidomain intervention programmes appear most informative when they are applied to enriched or at-risk populations rather than to unselected samples (Masserini et al., 2023).

Recent evidence suggests that structured multidomain interventions can improve global cognition in older adults at elevated risk, although the magnitude and domain distribution of benefit remain variable (Baker et al., 2025). Digital or lower-intensity multidomain approaches also suggest that mechanistic engagement may still be meaningful even when short-term cognitive effects are modest, especially in earlier-stage or prevention-oriented settings (Brodaty et al., 2025; Demnitz-King et al., 2025). This is relevant to convergence because physiological benefit may precede clear cognitive separation.

By contrast, anti-inflammatory or biologically targeted approaches may fail to show cognitive benefit for several reasons: the wrong inflammatory programme may have been targeted, impairment may already be too advanced, or the selected endpoint may be too distal from the mechanism under study (Mendes et al., 2025). A practical implication is that studies should avoid inferring success or failure from blood markers alone. If convergence is the mechanism being tested, demonstrating engagement of an NVU-relevant endpoint remains important. Conversely, a physiological shift in reserve or coupling in the absence of short-term cognitive change may still represent a meaningful biological signal.

#### 6.2.3 Preclinical and mechanistic evidence

Cellular and animal studies provide important bridges by showing that inflammatory signalling can alter endothelial phenotype, BBB transport regulation, glial activation states, and coupling-related pathways (Kempuraj et al., 2024). These data support the plausibility of bidirectional amplification, but translation remains constrained by limited multimorbidity, imperfect age equivalence, and incomplete correspondence between animal tasks and human cognitive domains.

Even so, preclinical work is particularly valuable when it predicts an intermediate phenotype that can be tested in humans. In this context, the most useful mechanistic studies are not those that merely show that inflammation affects the brain, but those that identify specific endothelial, glial, or flow-related consequences that can be operationalised with multimodal biomarkers.

Taken together, the literature supports convergence as a coherent and testable framework, but current human evidence remains stronger for integrative plausibility than for fully validated interaction models.

### 6.3 Operationalising convergence: a two-axis model

**Figure 3:**
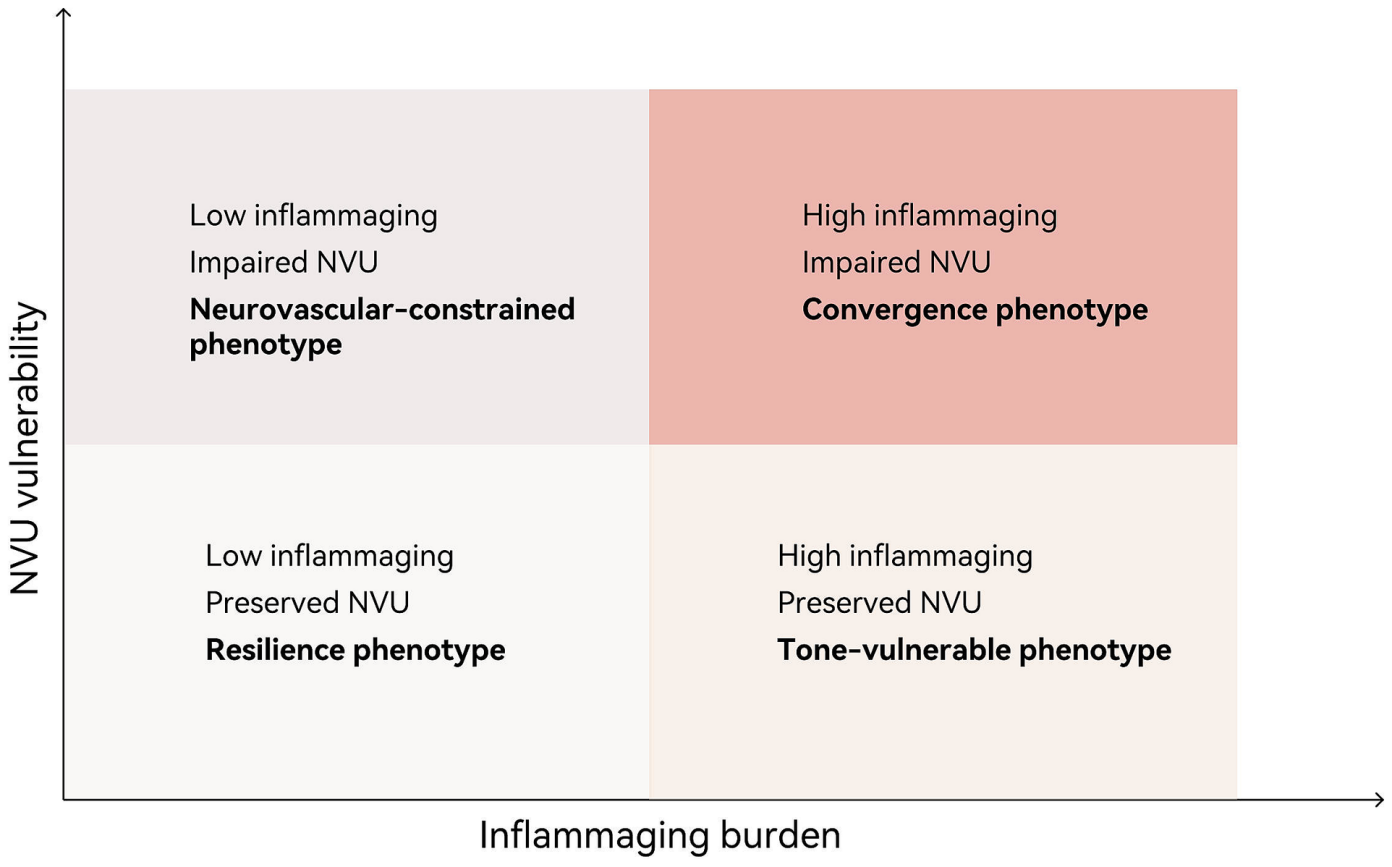
Two-axis framework for biomarker stratification and phenotype-informed therapeutic logic in cognitive ageing. Axis 1 represents inflammaging burden and Axis 2 represents neurovascular unit (NVU) vulnerability, indexed by reserve/coupling impairment together with BBB selectivity/transport dysfunction. The four quadrants are heuristic and continuous rather than rigid categories and illustrate how dominant biology, biomarker emphasis, and therapeutic priorities may differ across phenotypes.

For translational purposes, ageing-related vulnerability can be organised within a two-axis framework defined by inflammaging burden and NVU vulnerability. The value of this framework is pragmatic rather than purely descriptive: it may help explain heterogeneity, guide biomarker enrichment, and clarify why similar interventions yield inconsistent results across mixed populations (Hansra et al., 2024; Ma et al., 2025). Figure 3 translates the convergence framework into a two-axis state space that may support phenotype-informed therapeutic logic and more interpretable endpoint selection (Bentvelzen et al., 2025).

#### 6.3.1 A four-quadrant phenotype map

The four quadrants can be interpreted as heuristic phenotypes. Low inflammaging / preserved NVU represents a resilience phenotype, in which maintenance of reserve and slow cognitive decline are more relevant than near-term cognitive gain. High inflammaging / preserved NVU represents a tone-vulnerable phenotype, in which reduction of chronic inflammatory drive may be prioritised while reserve and coupling are monitored for early transition (Cui et al., 2024). Low inflammaging / impaired NVU represents a neurovascular-constrained phenotype, in which vascular optimisation and support of reserve/coupling are likely to be more central than immune modulation (Hosoki et al., 2023). High inflammaging/ impaired NVU represents a convergence phenotype, in which both axes are adverse and combined or sequenced strategies are most plausible (Lowry et al., 2021). These quadrants should not be interpreted as rigid categories, but as heuristics for thinking about dominant biology, likely effect modification, and reasonable expectations for treatment response (Wu et al., 2024). The translational implications of the four heuristic phenotype quadrants are summarised in Table 3.

#### 6.3.2 Why the two-axis model improves interpretability

A high inflammatory signal does not necessarily imply imminent cognitive decline if NVU buffering capacity remains preserved, and an NVU abnormality may remain clinically silent until inflammatory tone or stress exposure reduces compensatory margin (Song et al., 2025). By separating immune tone from buffering capacity, the model helps explain why effects dilute in unstratified cohorts and why apparently inconsistent findings may still be biologically compatible with the same underlying framework (Lennon and Sachdev, 2026).

The framework also clarifies why apparently negative studies may still be informative. An intervention may fail not because the targeted mechanism is irrelevant, but because the wrong phenotype was enrolled, the wrong outcome was emphasised, or the coupled axis remained unmeasured (Ip et al., 2024). This interpretive value is one of the model’s practical strengths even before it is fully validated.

#### 6.3.3 Implications for timing and testing

In earlier states, single-axis strategies may be sufficient to slow transition into the high–high quadrant. Once both axes are adverse, interventions may need to demonstrate engagement on both axes before meaningful cognitive change can be expected. This logic supports tiered biomarker panels, enriched enrolment, and greater reliance on intermediate physiological endpoints when short-term cognition is unlikely to shift (Wan et al., 2023).

Importantly, this does not imply that every future study must use complex multimodal panels. Rather, it suggests that the chosen markers should be adequate to the biological claim being made. If the claim is convergence, then both axes require meaningful representation.

## 7 Biomarkers

### 7.1 Principles for biomarker selection in an inflammaging–NVU framework

Within a convergence framework, biomarkers should be selected for function rather than novelty. Measures may be useful for at least four purposes: placing individuals within the two-axis space, demonstrating biological engagement in response to intervention, clarifying discordance between biology and cognition over short windows, and staging cumulative burden to inform expectations about reversibility (Badji et al., 2023; Hosoki and Sachdev, 2024).

Three principles follow. First, measures should be mapped to explicit constructs rather than treated as interchangeable. Second, longitudinal interpretability is more important than cross-sectional significance. Third, panels should represent both axes and support testing of interaction or effect modification (Biesbroek and Biessels, 2023; Tao et al., 2025).

These principles favour a tiered strategy: scalable blood signatures for repeated monitoring, function-proximal NVU physiology for stratification and intermediate endpoints, and slower structural or CSF measures deployed selectively to support mechanistic inference and staging (Wu et al., 2024; Liu et al., 2022). A further practical principle is proportionality. Not every study requires the same panel depth. Population studies may need scalable first-pass measures, whereas mechanistic sub-studies or early-phase trials may justify more invasive or complex multimodal phenotyping.

### 7.2 Blood-based markers

Blood-based biomarkers are attractive because they are scalable, repeatable, and suitable for longitudinal use. Within this framework, the aim is not to identify a single “best” marker, but to assemble interpretable signatures that reflect chronic inflammatory tone and vascular or endothelial stress relevant to NVU function. Repeated measures, composite scoring, and multimodal interpretation are preferable to one-off single-marker inference (Wan et al., 2023).

#### 7.2.1 Inflammaging-oriented signatures

Composite inflammatory indices may better capture persistent immune tone than isolated cytokine levels. Such signatures are most informative when related to cognitive slope over time, especially in executive and processing-speed domains. Their interpretation should prioritise stability and persistence rather than isolated elevation (Rao et al., 2026).

In practice, the value of these markers often lies less in absolute specificity than in repeated contextualised measurement. A modest but persistent inflammatory signal may be more informative than a large but transient excursion. Composite panels also reduce overreliance on any one molecule whose levels may be shaped by acute behaviours, intercurrent illness, or assay variation (Ma et al., 2024).

#### 7.2.2 Endothelial activation and vascular stress signatures

Blood markers reflecting endothelial activation, vascular inflammation, or haemostatic stress may provide peripheral windows onto neurovascular vulnerability. These measures are not BBB biomarkers per se, but they can help characterise the systemic vascular context in which BBB selectivity and coupling operate. Their value increases when interpreted alongside function-proximal or imaging-based NVU measures, such as cerebrovascular reactivity and BBB permeability-related imaging (Jaime Garcia et al., 2025).

This distinction is important because endothelial-state markers are often overinterpreted as direct surrogates for brain pathology. In the convergence framework, their role is more modest but still useful: they help estimate whether the systemic vascular milieu is likely to magnify the consequences of inflammaging for the NVU.

#### 7.2.3 Practical interpretation

Blood measures alone rarely place an individual precisely within the convergence framework, but they can support first-pass stratification and repeated monitoring. High inflammatory tone together with endothelial or vascular stress suggests a greater likelihood of a high–high phenotype, whereas discordant patterns should prompt deeper physiological or imaging-based characterisation. Blood markers are therefore best interpreted as anchors rather than arbiters.

### 7.3 CSF and other brain-proximal fluid markers

CSF and related brain-proximal fluids can strengthen inference by sampling a compartment closer to NVU and neuroimmune processes than blood. Their value lies not in automatic specificity, but in helping distinguish peripheral inflammatory signal from brain-relevant immune and barrier-associated activity (Hazan et al., 2023).

CSF is particularly informative when peripheral inflammatory tone is elevated but brain relevance remains uncertain, when imaging suggests NVU vulnerability despite low blood signal, or in mechanistic studies where central target engagement is of interest (Vrillon et al., 2025). Its use is constrained by invasiveness, limited scalability, and pre-analytical variability, so it is best positioned as a bridge modality rather than as a universal requirement.

Within the convergence framework, CSF may be most useful not as a stand-alone layer but as a tie-breaker in discordant cases. When blood and physiology point in different directions, or when peripheral inflammation and cognitive phenotype appear mismatched, brain-proximal sampling can sharpen inference about whether local neuroimmune amplification is present (Hong et al., 2025).

### 7.4 Neuroimaging and physiological markers of NVU function

Neuroimaging and vascular physiology provide the most direct means of operationalising the NVU axis by quantifying BBB-related dysfunction, perfusion efficiency, vascular reserve, neurovascular coupling, and cumulative structural burden. A recurrent pitfall is construct ambiguity: different methods index related but non-identical properties and remain sensitive to acquisition, preprocessing, and physiological state.

#### 7.4.1 Barrier-related imaging and transport proxies

BBB-oriented measures should distinguish permeability-related approaches from exchange- or transport-sensitive approaches. Dynamic contrast-enhanced MRI can provide permeability-related readouts (Montagne et al., 2015), whereas emerging tracer-free and water-exchange-based methods may offer complementary information regarding earlier barrier-related dysfunction (Lin et al., 2018). In selected settings, the CSF/serum albumin quotient (QAlb) and related fluid-based measures may provide additional barrier-associated information (Hillmer et al., 2023). These readouts are complementary rather than interchangeable.

In ageing, modest BBB alterations may be regionally specific and functionally relevant before frank “breakdown” is apparent (Preis et al., 2024). This distinction is important for the present framework: methods sensitive only to overt leak may systematically under-detect earlier selectivity or transport-related dysfunction. Conversely, claims that transport-oriented measures are superior require careful construct validation rather than assumption (Padrela et al., 2024; Padrela et al., 2025).

#### 7.4.2 Perfusion and microvascular efficiency

Arterial spin labelling (ASL) MRI and related perfusion approaches provide accessible measures of baseline cerebral blood flow. Their value lies primarily in characterising perfusion context and microvascular efficiency rather than in classifying individuals solely as having low or high resting perfusion. Perfusion measures are especially informative when combined with challenge-based or coupling-related approaches that assess whether baseline supply can be flexibly adjusted to demand (Huang et al., 2023).

#### 7.4.3 Cerebrovascular reactivity and vascular reserve

Cerebrovascular reactivity can be assessed using standardised physiological challenges, including CO₂-based paradigms or breath-hold protocols, together with MRI, transcranial Doppler, or other validated haemodynamic readouts. These approaches quantify the capacity of cerebral vessels to respond to physiological stress and are therefore well suited to the convergence model. Reduced reserve offers a mechanistic explanation for the disproportionate cognitive impact of inflammatory or haemodynamic stressors in older adults (Potvin-Jutras et al., 2025; Sleight et al., 2021).

Protocol harmonisation is essential because cerebrovascular reactivity measures are sensitive to challenge design, baseline CO₂, blood pressure, medication exposure, and analytical choices.

#### 7.4.4 Neurovascular coupling measures

Neurovascular coupling can be operationalised using task-linked haemodynamic paradigms, including task-based functional MRI, functional near-infrared spectroscopy (fNIRS), or Doppler-based approaches where appropriate. These measures are most informative when neural demand and haemodynamic response are interpreted together. Their value is maximised in longitudinal designs and when analysed alongside inflammatory tone. Standardisation remains challenging, but their conceptual importance is high because they lie at the interface between vascular physiology and network function (Owens et al., 2024).

#### 7.4.5 Structural NVU footprints

Structural MRI markers, including white-matter hyperintensity burden on fluid-attenuated inversion recovery (FLAIR) imaging and diffusion-based white-matter measures, provide cumulative records of neurovascular stress. Within the convergence model, these markers are best viewed as downstream footprints and stage markers rather than as direct readouts of current NVU function. Additional clearance-adjacent imaging readouts, including perivascular signal proxies, may eventually enrich this layer, although construct interpretation remains an area of active debate (Solé-Guardia et al., 2025).

### 7.5 Putting biomarkers together: a minimal convergence panel

A pragmatic convergence panel should support baseline positioning, early biological readout, and staging. At minimum, it should include: (i) a repeated blood-based immune-tone composite; (ii) a peripheral endothelial or vascular stress signature; and (iii) at least one function-proximal NVU endpoint, such as cerebrovascular reactivity, vascular reserve, or coupling-related physiology, optionally complemented by perfusion measures. Structural markers are best used for staging and prognosis, whereas CSF measures may be reserved for mechanistic sub-studies or discordant cases.

This minimum panel does not resolve all measurement challenges. Rather, it establishes a practical floor below which convergence claims become difficult to interpret. Minimum measurement sets and endpoint priorities for different observational and interventional study designs are summarised in Table 1.

**Table 1:** Minimum measurement sets and endpoint priorities for testing the inflammaging–NVU convergence framework in cognitive ageing.

Study aim/use case	Minimum inflammaging measures	Minimum NVU measures	Preferred intermediate endpoints	Cognitive/structural outcomes	Main interpretive pitfalls
Baseline stratification in observational cohorts	Repeated inflammatory composite; optional endothelial or vascular stress signature	At least one function-proximal NVU measure: CVR, using a standardised CO₂-based or breath-hold protocol where appropriate, or neurovascular coupling, using a task-linked haemodynamic approach; optional BBB selectivity/transport metric	Baseline two-axis positioning; identification of discordant phenotypes	Domain-specific cognition, especially processing speed and executive function; white-matter burden where available	Single time-point blood sampling may misclassify chronic immune tone; one-off NVU measures may be physiologically labile
Longitudinal testing of temporal ordering	Repeated inflammatory composite across multiple time points; immune-cell phenotyping where feasible	Repeated CVR and/or coupling measures; BBB selectivity/transport metrics where feasible; ASL or other validated perfusion measures as supportive context	Change in inflammaging burden; change in reserve/coupling; emergence of BBB selectivity/transport abnormality	Cognitive slope over time; progression of white-matter or small-vessel injury markers	Sparse sampling may obscure temporal ordering; null findings may reflect timing mismatch rather than absence of convergence
Early-phase mechanistic intervention studies	Pre/post inflammatory composite; target-relevant endothelial or vascular stress markers	At least one mechanism-proximal NVU endpoint, preferably CVR or task-linked coupling; optional BBB-related physiological metric, such as a permeability-related or exchange-sensitive imaging measure	Early biological engagement on the targeted axis; improvement in reserve/coupling and/or reduction in inflammatory burden	Short-term domain-specific cognition as a supportive outcome; structural measures mainly for staging	Blood-marker change alone may overstate success; short follow-up may miss downstream cognitive benefit
Phenotype-enriched proof-of-concept trials	Repeated inflammatory composite plus endothelial or vascular stress signature	CVR and/or task-linked coupling; BBB selectivity/transport metric where feasible; structural staging marker, such as white-matter hyperintensity burden or a diffusion-based white-matter measure	Dual-axis biological engagement; shift in the intended limiting axis, or both axes in convergence phenotypes	Domain-specific trajectories; white-matter stability or progression; global cognition as a secondary outcome	Incorrect phenotype assignment may dilute treatment effects; advanced structural burden may limit reversibility
Scalable population studies with limited imaging depth	Repeated inflammatory composite; optional endothelial or vascular stress signature	One scalable NVU-oriented proxy, preferably CVR, ASL or another validated perfusion measure, or a validated reserve-related physiological measure	Risk enrichment; coarse two-axis placement	Repeated cognitive testing; structural MRI markers where feasible	Reduced construct specificity; absence of BBB-selective or coupling measures limits mechanistic inference

“Minimum” refers to the smallest set of measures likely to permit interpretable testing of the inflammaging–NVU convergence framework, rather than an optimal or exhaustive panel. Across study types, at least one repeated inflammatory measure and one function-proximal NVU measure are preferred if convergence is the biological claim being tested. BBB selectivity/transport measures are especially informative where feasible. Representative techniques are provided as operational examples rather than mandatory protocols; their selection should depend on the construct under investigation, feasibility, and protocol validation. ASL, arterial spin labelling; BBB, blood–brain barrier; CVR, cerebrovascular reactivity; MRI, magnetic resonance imaging; NVU, neurovascular unit.

**Table 2:** Key inflammatory–neurovascular unit (NVU) interfaces in cognitive ageing: representative programmes, predicted consequences, human readouts, evidentiary strength, and major uncertainties.

Mechanistic interface	Representative inflammatory programmes	Predicted NVU consequences	Representative human readouts and operational examples	Likely cognitive correlates	Current evidentiary strength	Major uncertainties
Immune–endothelium	Endothelial-activating cytokine and redox–inflammatory programmes	Endothelial activation; reduced NO bioavailability; impaired BBB selectivity/transport; reduced vasoreactivity	Endothelial and vascular stress markers; BBB-oriented imaging, including DCE-MRI for permeability-related readouts and exchange-sensitive or water-exchange approaches where feasible; CVR assessed using standardised CO₂-based or breath-hold protocols; ASL or other validated perfusion measures	Processing speed; executive dysfunction; reduced physiological stress tolerance	Moderate human associative evidence; stronger mechanistic support from preclinical studies	Causal direction; peripheral-to-central mapping; distinction between selectivity/transport dysfunction and overt leak
Glia–vascular	Innate immune amplification, inflammasome-linked signalling, and complement programmes	Microglial priming; astrocytic endfoot remodelling; impaired vascular support; sustained local amplification	Multimodal inflammatory markers; brain-proximal fluid markers where feasible; coupling-related physiology, including task-linked fMRI, fNIRS, or Doppler-based approaches where appropriate; white-matter and network-level signatures	Network inefficiency; cognitive fatigability; mixed-domain dysfunction	Limited direct human integrative evidence; substantial mechanistic plausibility	Peripheral-to-central correspondence; regional heterogeneity; reversibility in humans
Flow–metabolic	Redox–inflammatory and glia–vascular signalling programmes	Impaired neurovascular coupling; reduced vascular reserve; dynamic supply–demand mismatch	CVR; neurovascular coupling measures; ASL or other validated perfusion measures; task-linked haemodynamic readouts	Executive dysfunction; attention; processing speed	Growing human physiological evidence; substantial methodological heterogeneity	Protocol harmonisation; temporal ordering; endpoint sensitivity

Representative supporting references are cited in the main text. Operational examples are illustrative rather than mandatory and should be selected according to the construct under investigation, feasibility, and protocol validation. Permeability-related and exchange-sensitive BBB measures are complementary rather than interchangeable. ASL, arterial spin labelling; BBB, blood–brain barrier; CVR, cerebrovascular reactivity; DCE-MRI, dynamic contrast-enhanced magnetic resonance imaging; fMRI, functional magnetic resonance imaging; fNIRS, functional near-infrared spectroscopy; NO, nitric oxide; NVU, neurovascular unit.

**Table 3:** Translational implications of the two-axis inflammaging–NVU framework in cognitive ageing.

Phenotype quadrant	Biological profile	Priority biomarkers for stratification	Most informative intermediate endpoints	Therapeutic emphasis	Main caveats/misclassification risks
Low inflammaging/preserved NVU	Low chronic immune tone; preserved neurovascular buffering capacity, reserve/coupling, and BBB selectivity/transport; slower expected progression	Repeated inflammatory composite; baseline CVR or neurovascular coupling measure; optional BBB selectivity/transport measure for mechanistic confirmation	Cognitive slope over time; stability of reserve/coupling; maintenance of white-matter integrity	Maintenance and prevention; vascular risk control; lifestyle optimisation; avoidance of overtreatment	Early subclinical change may be missed because the phenotype appears biologically “normal”; low short-term event rates may obscure future risk
High inflammaging/preserved NVU	Elevated chronic immune tone with relatively preserved neurovascular buffering capacity and reserve/coupling	Repeated inflammatory composite; endothelial or vascular stress signature; baseline CVR or coupling measure to detect transition risk	Reduction in inflammatory burden; early decline in reserve/coupling; emerging BBB selectivity/transport abnormality	Immune-tone modulation; upstream anti-inflammatory and lifestyle strategies; close monitoring for transition to NVU vulnerability	Peripheral inflammatory signals may not reflect brain relevance; transient inflammatory states may mimic stable inflammaging burden
Low inflammaging/impaired NVU	Modest systemic inflammatory burden but reduced neurovascular buffering capacity, impaired reserve/coupling, and/or BBB selectivity/transport dysfunction	CVR; neurovascular coupling; ASL or other validated perfusion measures; BBB selectivity/transport metrics; structural small-vessel markers for staging	Improvement in reserve/coupling; better perfusion efficiency; BBB-related physiological change	NVU-directed support; endothelial optimisation; reserve restoration; coupling-oriented strategies	Low blood inflammatory signals may falsely imply low biological risk; vascular and neurodegenerative contributions may be difficult to disentangle
High inflammaging/impaired NVU	Coupled vulnerability with elevated immune tone and reduced neurovascular buffering capacity; greatest likelihood of amplification loops	Repeated inflammatory composite; endothelial or vascular stress markers; CVR and/or coupling measures; BBB selectivity/transport metrics; structural staging markers	Dual-axis biological engagement; improvement in reserve/coupling and inflammatory burden before expecting cognitive gain	Combined or sequenced immune- and NVU-directed strategies; phenotype-enriched intervention designs	Greatest heterogeneity and misclassification risk; advanced structural burden may limit reversibility; null short-term cognitive change does not exclude biological engagement

The quadrants are heuristic and continuous rather than rigid categories. They are intended to support biomarker stratification, intermediate endpoint selection, and interpretation of heterogeneous intervention responses, rather than fixed clinical classification. ASL, arterial spin labelling; BBB, blood–brain barrier; CVR, cerebrovascular reactivity; NVU, neurovascular unit.

## 8 Therapeutic opportunities

### 8.1 Therapeutic logic: matching interventions to the two-axis state space

The convergence framework suggests that treatment effects may depend importantly on baseline position within the two-axis space. The quadrants should be understood as states with possible transitions rather than static labels. Therapeutic aims are therefore twofold: to prevent transition into a high–high convergence state by stabilising the vulnerable axis early, and to weaken amplification loops once both axes have become adverse (Lin et al., 2025).

In practice, low inflammaging / preserved NVU phenotypes are more likely to benefit from maintenance and resilience-oriented strategies than from approaches expecting near-term cognitive gain. High inflammaging/preserved NVU phenotypes may represent an early window for immune-tone modulation. Low inflammaging/impaired NVU phenotypes are more plausibly dominated by reduced neurovascular buffering capacity. High inflammaging/impaired NVU phenotypes are the clearest candidates for dual-axis approaches. This two-axis framework is most useful when translated into intervention strategies that either reduce inflammatory load, restore NVU buffering capacity, or engage both axes when vulnerability is coupled.

### 8.2 Anti-inflammatory strategies

Anti-inflammatory therapy is an intuitively attractive response to inflammaging, but the term encompasses mechanistically diverse approaches. The central question is whether a given strategy modulates programmes that meaningfully constrain NVU function rather than merely changing peripheral markers (Li et al., 2025a).

#### 8.2.1 Targeting upstream amplification and demonstrating brain relevance

Upstream approaches that attenuate amplification loops, including maladaptive innate activation or senescence-associated inflammatory signalling, may be more relevant to persistent immune tone than downstream suppression of individual markers (Suda et al., 2024; Chen P. et al., 2024). Lowering a single cytokine does not necessarily imply improved endothelial function or restored coupling. A recurrent translational pitfall is the demonstration of systemic anti-inflammatory effects without evidence of impact on NVU physiology or central immune state. Biological engagement should therefore be assessed using mechanism-aligned readouts, such as endothelial function, BBB-related physiology, neuroimmune markers, or coupling-related measures.

#### 8.2.2 Timing and heterogeneity

Null findings in unstratified late-life samples should be interpreted cautiously, as they may reflect late intervention, insufficient target engagement, or dilution of effect across mismatched phenotypes rather than true irrelevance of inflammatory biology (Masserini et al., 2025).

#### 8.2.3 Safety constraints

In older adults, immunomodulation is constrained by infection risk, impaired vaccine response, comorbid organ dysfunction, and polypharmacy. These realities favour approaches that restore immune balance without indiscriminate suppression, especially when paired with strategies that support NVU function directly (Dove et al., 2024).

### 8.3 NVU-targeted strategies

NVU-directed approaches aim to stabilise vascular homeostasis and buffering capacity, thereby reducing vulnerability to both chronic inflammaging and transient systemic stressors (Duve et al., 2025).

#### 8.3.1 Endothelial support

Interventions that improve endothelial health and vasoactive balance are attractive because endothelial dysfunction plausibly links inflammaging to impaired reserve and coupling. Their translational value depends on demonstrating measurable improvement in cerebral reserve, BBB-related function, or coupling, rather than only favourable changes in systemic cardiovascular risk profiles (Guse et al., 2025).

#### 8.3.2 Reserve restoration and microcirculatory efficiency

Because reduced reserve amplifies the cognitive impact of stressors, approaches that increase reserve may function as buffer restorers. These are especially relevant in NVU-constrained phenotypes and may synergise with inflammatory tone reduction in convergence states (Stern et al., 2019).

#### 8.3.3 Coupling repair

Approaches that improve neurovascular coupling are attractive because they target dynamic supply–demand mismatch close to cognition. However, because coupling is an emergent NVU property, effective interventions may need to act on multiple components rather than a single pathway (Zhu et al., 2022).

Their translational promise lies partly in timing: coupling may change earlier than cognition or structural burden, making it a useful bridge outcome in mechanistic studies and early-phase trials.

### 8.4 Combination and sequencing

The convergence framework predicts that clinically meaningful benefit may require dual-axis engagement, particularly once amplification loops are established. In some settings, sequencing may be more practical than concurrent combination therapy, with immune-directed strategies used earlier when reserve is preserved and NVU-directed support prioritised when reserve or coupling is already impaired (Xu et al., 2025; Sugimoto et al., 2024). The value of combination or sequencing depends on whether the biology truly supports coupled vulnerability and whether each component can be meaningfully monitored.

### 8.5 Practical barriers

Three constraints dominate translation: multimorbidity, polypharmacy, and endpoint mismatch. Multimorbidity confounds both inflammatory and NVU biomarkers; polypharmacy limits tolerability and complicates attribution; and cognition often changes more slowly than the biological systems of interest. These considerations reinforce the case for enriched phenotyping and for pairing cognitive outcomes with mechanism-aligned biological endpoints (Soldevila-Domenech et al., 2025).

## 9 Future directions and conclusions

### 9.1 Research priorities

To move from an integrative framework to an actionable and testable model, the field needs designs that resolve interaction, timing, and treatability.

First, longitudinal multimodal studies should explicitly test whether immune tone predicts cognitive decline preferentially in NVU-vulnerable phenotypes.

Second, NVU-relevant measures require clearer construct validation and harmonisation, especially the distinction between BBB selectivity/transport and permeability, and between reserve/reactivity and coupling (The VASCOG-2-NP Consortium, 2025).

Third, the field would benefit from minimal reproducible biomarker panels that represent both axes and can be deployed across cohorts.

Methodological fragmentation remains a major barrier. Studies often measure inflammatory markers, vascular physiology, BBB proxies, or cognition in isolation, using different timescales and construct definitions. Harmonisation will therefore be as important as biological innovation if convergence is to become a practical framework rather than a compelling but under-tested idea (Lim et al., 2025).

Fourth, mechanism-aligned trials should incorporate early biological readouts alongside cognition, particularly where short-term cognitive change is unlikely (Hosoki and Sachdev, 2025).

Fifth, future work should define therapeutic windows more clearly, distinguishing potentially reversible functional NVU impairment from later-stage structural burden.

Emerging single-cell, spatial transcriptomic, and multiplex tissue-imaging approaches may also help refine the framework by resolving cell-type-specific and region-specific inflammatory–vascular interactions within the ageing brain and its border tissues (Yuan et al., 2024; Duhan et al., 2024). These methods are unlikely to provide immediate clinical biomarkers, but they may help clarify which endothelial, glial, and mural-cell states correspond most closely to the phenotypes described here and may thereby sharpen future translational models.

A further priority is the integration of stressor-based designs. Because the convergence model predicts disproportionate effects of transient perturbation in low-reserve, high-inflammation phenotypes, carefully measured challenge paradigms may reveal vulnerabilities that remain obscure under resting conditions alone (He et al., 2025).

## 10 Conclusion

Current evidence suggests that age-related cognitive decline is often better understood as a systems problem in which inflammaging and NVU dysfunction converge to erode buffering capacity, destabilise neurovascular coupling, and lower the threshold for clinical expression of mixed pathologies. The convergence model generates testable predictions: cognitive trajectories should depend importantly on the joint state of immune tone and NVU reserve/coupling, and therapeutic benefit should be greatest when interventions either shift the dominant limiting axis early enough to prevent transition into a high-risk convergence state or engage both axes once convergence is established.

The model also admits clear falsifiers. If, under harmonised measurement and appropriate stratification, cognitive and NVU improvement repeatedly occurs without measurable engagement of the hypothesised axis, or if one-axis engagement reliably improves cognition without any corresponding change in the coupled immune or NVU domain, then the convergence framework would require revision. Progress will therefore depend on biomarker strategies and study designs that prioritise construct clarity, stratification, timing, and mechanism-aligned endpoints, with the ultimate aim of enabling safer and more targeted interventions for heterogeneous older populations.
